# Superior Frontal Sulcus Focal Cortical Dysplasia Type II: An MRI, PET, and Quantified SEEG Study

**DOI:** 10.3389/fneur.2019.01253

**Published:** 2019-12-03

**Authors:** Chao Zhang, Bao-tian Zhao, Aileen McGonigal, Wen-han Hu, Xiu Wang, Xiao-qiu Shao, Yan-shan Ma, Jian-guo Zhang, Kai Zhang

**Affiliations:** ^1^Departments of Neurosurgery, Beijing Tiantan Hospital, Capital Medical University, Beijing, China; ^2^Department of Functional and Stereotactic Neurosurgery, Beijing Neurosurgical Institute, Capital Medical University, Beijing, China; ^3^INSERM UMR 1106, Institut de Neurosciences des Systèmes, Marseille, France; ^4^Faculty of Medicine, Aix-Marseille University, Marseille, France; ^5^Clinical Neurophysiology Department, Timone Hospital, Assistance Publique des Hôpitaux de Marseille, Marseille, France; ^6^Departments of Epilepsy, Beijing Tiantan Hospital, Capital Medical University, Beijing, China; ^7^Department of Neurosurgery, Beijing Fengtai Hospital, Beijing, China

**Keywords:** superior frontal sulcus, focal cortical dysplasia, semiology, epileptogenic zone, epileptic network

## Abstract

**Purpose:** The superior frontal sulcus (SFS), located in the prefrontal and premotor cortex, is considered as one of the common locations of focal cortical dysplasia (FCD). However, the characteristics of seizures arising from this area are incompletely known. The primary purpose of this study was to investigate the clinical features and the epileptic networks of seizures originating from the SFS.

**Methods:** We included seventeen patients with type II FCD within the SFS. SFS was identified both visually and automatically. Semiological features were evaluated and grouped. Interictal 18FDG-PET imaging in all patients was compared to controls using statistical parametric mapping (SPM-PET). In those subjects with stereoelectroencephalography (SEEG), two different quantitative intracranial electroencephalography analyses were applied. Finally, the locations of the SFS-related hypometabolic regions and epileptogenic zones (EZs) were transformed into standard space for group analysis.

**Results:** We identified two semiological groups. Group 1 (9/17) showed elementary motor signs (head version and tonic posturing), while group 2 (8/17) exhibited complex motor behavior (fear, hypermotor, and ictal pouting). Based on SPM-PET, an SFS-supplementary motor area (SMA) epileptic propagation network was found in group 1, and an SFS-middle cingulate cortex (MCC)-pregenual anterior cingulate cortex (pACC) propagation network was discovered in group 2. Intracranial EEG analysis suggested similar affected structures with high epileptogenicity. The SFS-related hypometabolic regions and EZs in these groups showed a posterior-anterior spatial relationship.

**Conclusions:** Even though originating from the spatially restricted cortex, SFS seizures can be divided into two groups based on semiological features. The SFS-SMA and SFS-MCC-pACC epileptic propagation networks may play pivotal roles in the generation of different semiologies. The posterior-anterior spatial relationship of both hypometabolic regions and EZs provides potentially useful information for distinguishing different types of SFS seizures and surgical evaluation.

## Introduction

Focal cortical dysplasia (FCD) is a common cause of refractory epilepsy (1). Despite high-resolution MRI, FCD may be challenging to detect radiologically; however, even in MRI-negative cases, the epileptogenic zone (EZ) associated with FCD may be localized using stereoelectroencephalography (SEEG) and/or interictal FDG-PET, allowing surgical treatment and good outcome in a high proportion (2). FCD, especially FCD type II, may often occur within frontal lobes (1, 3, 4), with a prefered location in the depth of sulci for small lesions (4). A recent study has shown that the semiology of frontal lobe seizures can be correlated with the anatomic organizations along with a rostrocaudal axis (5). However, the detailed semiological patterns of seizures originating from superior frontal sulcus (SFS) as well as their underlying epileptic networks remain incompletely elucidated, contributing to a non-negligible failure rate in frontal lobe epilepsy surgery, especially for MRI-negative cases (6).

Anatomically, the SFS divides superior and middle frontal gyri and its morphology changes across individuals and human lifespan (7). SFS has been considered as one of the most common sites of FCD (8). Early SEEG studies by Bancaud and Talairach emphasized the occurrence of tonic head and eye version as well as visual illusions and hallucinations, forced thinking, complex postural signs, and frequent secondary generalization during seizures from the dorsolateral prefrontal convexity (9). Due to the complexity of semiological manifestations, intracranial EEG may often be required, especially when MRI is negative. SEEG presents advantages in this scenario, allowing exploration of both superficial and deep frontal structures; thus, the base of a sulcus, a common site for FCD (4), can be readily explored. However, no previous studies have characterized the spectrum of semiology and underlying FCD type II–related epileptic networks centered around the SFS.

In this study, we retrospectively included a cohort of epilepsy patients who had EZ within the SFS, which were determined by SEEG results and/or seizure freedom after surgery. Both manual and automatic sulci identification on MRI was performed to identify the SFS (10). To achieve better consistency, we enrolled only patients who exhibited the pathological manifestation of FCD type II. We firstly analyzed the semiological features, based on which the patients were grouped in 2 subtypes. To explore the underling epileptic network, we analyzed interictal hypometabolism with 18F-fluorodeoxyglucose (18FDG-PET). In those subjects with ictal SEEG, we conducted two different quantitative intracranial electroencephalography analyses.

## Materials and Methods

### Patient Selection

This is a retrospective study performed in Beijing Tiantan Hospital, Epilepsy Center. From our epilepsy surgery database, we initially enrolled 87 consecutive frontal lobe epilepsy patients who underwent resective surgery and exhibited histologically proven FCD type II from January 2015 to December 2018. To ensure that the FCD lesions or the EZs were located within the SFS, we first identified the SFS both visually and automatically (detailed below). For inclusion, the following criteria also needed to be met: for direct surgery, the FCD lesions had to overlap the SFS strictly, or most of the FCD had to lie within the SFS; for stereoelectroencephalography (SEEG) cases, the FCD lesions needed to overlap the SFS strictly, and the SEEG electrode contacts that maximally involved at seizure onset needed to be located within the SFS. The Ethics Board of Beijing Tiantan Hospital approved this study and written informed consent was obtained from all participants.

### Image Acquisition

MRI scans were performed on a 3T Siemens Verio scanner (Siemens Medical System, South Iselin, NJ), including a 3D-T1 sagittal magnetization prepared rapid gradient echo sequence (MPRAGE, TR/TE 1900/2.53, TI 900, matrix 256 × 256, 1.0 mm thickness), T2 axial (TR/TE 7030/110, matrix 256 × 320, 3 mm thickness), FLAIR axial (TR/TE 8000/94, TI 2371.5, matrix 424 × 512, 3 mm thickness), FLAIR sagittal (TR/TE 8000/96, TI 2371.2, matrix 236 × 256, 3 mm thickness), and FLAIR coronal (TR/TE 8000/96, TI 2371.2, matrix 408 × 512, 3 mm thickness) imaging. The 18FDG-PET examination was performed using a GE Discovery ST PET-CT system (300 mm FOV, matrix 192 × 192, and 3.27 mm slice thickness). An IV injection of 18FDG at a mean dose of 220 MBq/70 kg body weight was administered. Reconstructed images were corrected for attenuation using transmission scans obtained from a germanium source. PET scans of all patients were obtained within 6 months before the epilepsy surgery evaluation. No patients had ictal events <6 h before or during the PET scanning.

### SFS Recognition

Visual identification of the SFS: two senior neurosurgeons (Kai Zhang and Jian-guo Zhang) reviewed the MRI 3D T1 images and identified the SFS independently, and disagreements were resolved through discussion within the study group.

Automatic recognition of the SFS: SFS was extracted and identified using 3D T1 MRI images through Morphologist 2015, an image processing pipeline integrated in BrainVISA (http://brainvisa.info). Briefly, the procedures included: (i) brain segmentation of the gray matter (GM), white matter (WM), and cerebrospinal fluid (CSF), (ii) reconstruction of the interface corresponding to the GM–WM and GM–CSF, (iii) extraction of sulci folds by segmenting the skeletonized GM–CSF interface into simple surfaces, and (iv) after the extraction, automatic recognition of the sulci through the statistical probabilistic anatomy map method (SPMA) (10).

### SEEG Recordings

Patients underwent intracranial evaluation independently of this study according to information available from the non-invasive study and clinical hypotheses about the localization of the EZ (11). The details of the intracranial multiple contact depth electrodes as follows: Beijing HKHS Healthcare co., ltd, China, 8-16 contact, length: 2 mm, diameter: 0.8 mm, 1.5 mm apart. A postoperative CT scan was applied to verify the absence of bleeding and the position of the electrode contacts. We used IntrAnat, a software based on BrainVISA, to coregister the CT to the MRI, precisely localized the electrode contacts by image processing, and prepared the database for subsequent studies (12). Video-EEG recording was prolonged as long as necessary in order to record several habitual seizures. SEEG signals were recorded using a Nihon Kohden video-EEG system (Neurofax EEG-1200, Nihon Kohden Corporation, Tokyo, Japan) with a sampling rate of 1,000 or 2,000 Hz. For the anatomical description, the subdivision of the frontal Brodmann areas is represented according to Petrides and Pandya's work (13). The cingulate cortex was divided based on Vogt's study (14).

### Semiology Analysis and Grouping

We retrospectively selected ictal symptoms and signs proven to be habitual and reproducible for analysis. Chao Zhang and Xiu Wang (both epileptologist and have over 5 years of experience in epilepsy presurgical evaluation) reviewed the ictal semiology and classified the patients independently (kappa value = 0.76), and all the results were discussed with a senior epileptologist (Xiao-qiu Shao). Seventeen SFS patients were divided into two groups. Group 1 was defined as those patients mainly manifested as elementary motor signs, and Group 2 showed a mixture of mainly proximal complex motor behavior and elementary motor signs (5). Based on such findings, we sought to examine whether there were two distinct types of epileptic networks that may explain the rationality of such a grouping result and its underlying mechanisms. To verify this hypothesis, we analyzed the data in the following three ways.

### PET Image Processing and SPM Analysis

The PET images were analyzed through SPM12 running on MATLAB (2018a). All the lesion-related PET images were lateralized to the left side for group analysis. First, the images were spatially normalized to the Montreal Neurological Institute atlas (voxel size: 1 mm × 1 mm × 1 mm) and then smoothed with a Gaussian filter (8 mm FWHM) to increase the signal-to-noise ratio. The smoothed images were divided by the mean global FDG value individually to control the variation between different samples. A brain mask not containing the scalp, cerebrospinal fluid and cerebellum was used to select voxel activities and to exclude extracranial activities. The parametric images were compared with those of a group of 18 healthy subjects (male: *n* = 10, age: 22.6 ± 3) using voxel-based independent *t*-test analysis as described in our previous study (15). A cluster threshold of 100 voxels was applied, with *P* < 0.001 and *P* values corresponding to the false discovery rate (FDR) correction.

### SEEG Ictal Recordings: Visual Analysis, Computation of the Epileptogenicity Index (EI), and Epileptogenicity Mapping (EM)

All seizure episodes recorded by SEEG were visually reviewed by two reviewers (Chao Zhang and Xiu Wang) and then discussed with a senior neurophysiologist. After the discussion, first, an agreement about the location of seizure onset and structures involved in the epileptic propagation was established, and then two habitual seizures were confirmed and collected for the following analysis (16, 17). Although patient 14 was suffering daily seizure before intracranial electrodes implantation, none of the ictal seizure was discovered after 1 month of monitoring.

The EI is a normalized quantitative method that computes the iEEG signal with the purpose of objectively quantifying and defining structures with high epileptogenicity underlying seizure propagation (18). In summary, the EI is intended to quantify two essential features of ictal activity: spectral (appearance of high-frequency oscillations replacing the background activity) and temporal (delay of appearance concerning seizure onset) properties. The EI is a normalized range from 0 (no epileptogenicity) to 1 (maximal epileptogenicity), and an EI value of 0.3 was set as the threshold to define a channel as epileptogenic or not (18). We computed the EI for all selected seizures by using a plugin function in AnyWave software, an open-access tool (http://meg.univ-amu.fr/wiki/AnyWave) (19). Parameters in the AnyWave software were adjusted in reference to the visual observation.

To obtain more detailed spatial information of structures with high epileptogenicity, we conducted another quantitative iEEG analysis, epileptogenicity mapping (EM) (20), which was based on the Statistical Parametric Mapping (SPM, version 12, www.fil.ion.ucl.ac.uk/spm) toolbox in MATLAB (2018a). Briefly, EM quantifies the *T*-value from the change in the iEEG amplitude in the involved high-frequency band by comparing interictal and early ictal periods (two-sample *t*-test). Here, we defined the frequency band of interest as 80–250 Hz. For the preictal baseline, a period time of at least 30 s was selected as the control, and a time window of 5 s was isolated for seizure onset. The EM for each patient was demonstrated after local interpolation of the *T*-value to produce an image superimposed on the individual MRI. For the group study, EM images were averaged and co-registered with the MNI-space template.

### Positional Relationship Analysis of the SFS-Related Hypometabolic Regions and High Epileptogenicity Areas Between the 2 Groups

In our preliminary observations, we assumed that there might be positional differences in the epileptic area between the two groups. To verify this hypothesis, we applied an analysis based on two perspectives as follows. From a functional perspective, based on the aforementioned PET/SPM method, we extracted the ROI around the SFS, which had passed the FDR correction, responding with *P* = 0.05. From the electrical epileptogenic aspect, based on the aforementioned EM method, an ROI related to the SFS showing a *T*-value >25 was selected. All the ROIs were stored in NIfTI format and then clustered into their respective centroids in MNI space by the built-in function of k-means in MATLAB. The Euclidean distance between the centroids in 2 groups was calculated. For a fair presentation of the results, all the ROIs according to centroids were converted into the 3D surface mesh in the VTK format in ITK-SNAP (v. 3.0.0; http://www.itksnap.org) and rendered in 3D superimposed with a glass brain image in MNI space in ParaView (v. 4.3.1; http://www.paraview.org) (21).

## Results

### Clinical Features and Semiology Classification: Elementary Motor Signs vs. Complex Motor Behavior

**Figure 1:**
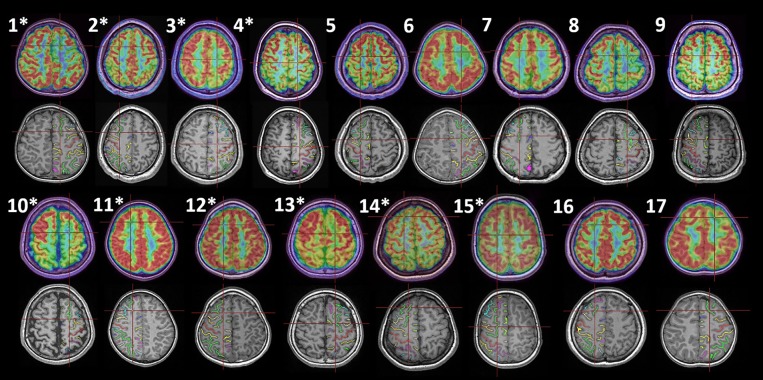
Histologically proven FCD type II in the SFS of 17 patients. The upper and lower rows are group 1 (1–9) and group 2 (10–17), and the asterisks indicate cases with SEEG evaluation. For each patient, two coregistration images (MRI-PET and MRI-automatically identified sulci, Morphologist 2015, BrainVISA) with an identical axial position are displayed. The intersection of the red line demonstrates the location of the electrode contact, which is considered to be the seizure onset (1–4, 10–15) or the most apparent lesion (5–9, 16, 17). The green sulcus in the frontal lobe is the automatically recognized SFS.

Seventeen SFS cases were included for analysis (Figure 1). In this series, nine were male, and the mean epilepsy duration was 7.9 ± 1.6 years. SEEG was conducted in 10/17 patients. Mean age at surgery was 15.8 ± 2.3 years. The surgical extent based on the post-surgery CT/MRI and the surgical plan was presented in Supplementary Materials. All cases were pathologically proven to be FCD type II, following the classification of Blumcke et al. (22). After 28.3 ± 3.3 months of follow-up, 16/17 patients were seizure-free (Engel Ia). The general characteristics and main clinical semiology are shown in Table 1.

**Table 1:** Demographic and clinical data of 17 SFS epilepsy patients.

**Group**	**No**.	**Gender**	**Age (years old)**	**Onset age (years old)**	**Seizure per month**	**Semiology**	**MRI**	**SEEG**	**Pathology**	**Outcome (Engel)**
1	1	M	4	1	500–600	Limbs tonic → R head version → limbs clonic	+	Y	FCD IIa	Class Ia
1	2	M	21	5	20–24	Upper limbs rising → head turning to R → soliloquizing	–	Y	FCD IIa	Class Ia
1	3	M	25	17	30–60	R head version → upper limbs rising → limbs tonic/clonic (asymmetric tonic posturing)	+	Y	FCD IIa	Class IIa
1	4	M	13	9	10–30	Upper limbs rising (asymmetric tonic posturing) → R head version	–	Y	FCD IIa	Class Ia
1	5	M	17	5	20–30	R upper limb rising/head turning to R → clonic of L limbs and facial	–	N	FCD IIa	Class Ia
1	6	F	3.4	0.4	250–300	Head/eyelids/upper limbs clonic	+	N	FCD IIb	Class Ia
1	7	F	35	14	30–100	L head version → upper limbs rising (asymmetric tonic posturing)	–	N	FCD IIb	Class Ia
1	8	F	23	5	250–300	R head version → upper limbs rising(asymmetric tonic posturing)	+	N	FCD IIb	Class Ia
1	9	M	22	14	30–60	Head turning to L/R arm flexion and repetitive movement → moaning occasionally	–	N	FCD IIa	Class Ia
2	10	M	16	5	30–90	Ictal pouting → R arm rising → moaning	+	Y	FCD IIb	Class Ia
2	11	F	10	6	30–300	Fear/heart rate increasing/flush → crying/holding hands	–	Y	FCD IIb	Class Ia
2	12	M	6	3	60–90	Fear/heart rate increasing/crying	–	Y	FCD IIa	Class Ia
2	13	M	12.25	12	90–150	Fear/heart rate increasing → R arm rising/tonic → pelvic movement/kicking/turning → shouting	–	Y	FCD IIb	Class Ia
2	14	F	28	19	30–60	R hand groping/ictal pouting → L head version	–	Y^*^	FCD IIb	Class Ia
2	15	F	7	6	300–400	Moaning and crying/head and trunk rocking left and right → laughing	–	Y	FCD IIa	Class Ia
2	16	F	22	9	10–15	Trunk rocking back and forth/twisting of shoulders, humming	–	N	FCD IIb	Class Ia
2	17	F	4.08	4	90–240	Fear, lower limbs paddling, stepping/crying	–	N	FCD IIa	Class Ia

*M, male; F, female; L, left; R, right; FCD, focal cortical dysplasia; +, MRI positive; –, MRI negative; Y, yes; N, no*.

* *Case 14 did not have any ictal seizure during 1 month of SEEG monitoring*.

Our cases showed highly stereotyped seizures. Based on video-EEG data, 9/17 patients were placed in group 1, in which 8/9 showed head version/turning and tonic posturing initially, and 4/9 expressed asymmetric tonic posturing in the middle of the ictal period. On the other hand, the remaining 8 patients showed mainly complex motor behavior. Four patients developed fearful expression in the early stage of seizure, and the other 4 revealed ictal pouting and proximal joint-based hypermotor activity. Two patients had limb elevation during the attack. Accordingly, these 8/17 cases were classified as complex motor behavior group.

### PET Data: Estimation of Hypometabolic Areas

**Figure 2:**
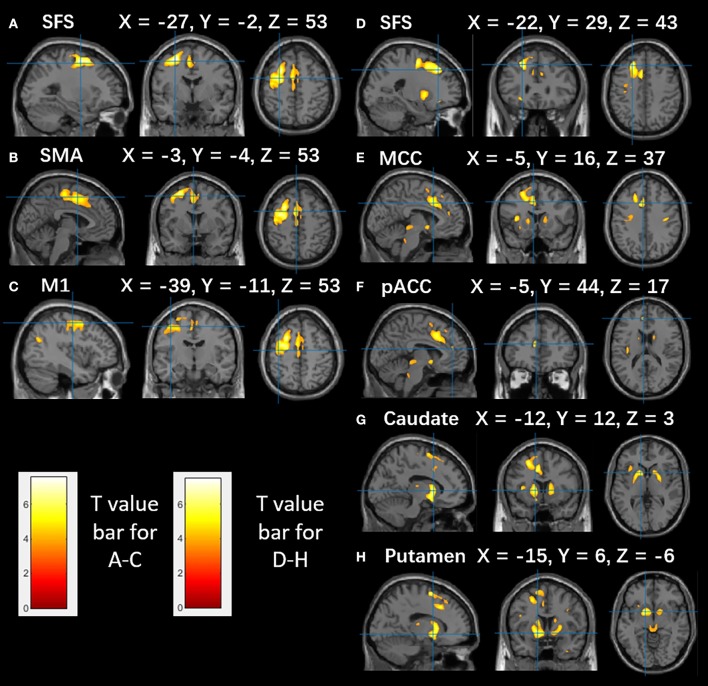
The metabolism of SFS epilepsy patients compared to that of healthy controls. Brain areas with decreased glucose metabolism are superimposed on the MNI152 template (*P* < 0.001). **(A–C)** Significant hypometabolism in the SFS, SMA, precentral gyrus in group 1. **(D–H)** Significant hypometabolism in the SFS, MCC, pACC, caudate, and putamen in group 2. The color scale indicates T scores. Note that PET images in patients with right-sided lesions are all horizontally flipped to the left side.

The difference between this cohort and the controls was calculated with thresholds of *P* < 0.001 and PFDR-cor < 0.05. When *P* < 0.001 was considered, decreased metabolism in group 1 was observed in SFS, supplemental motor area (SMA), and the superior portion of primary motor cortex (M1) (Figures 2A–C). Group 2 showed significant hypometabolism in the SFS, MCC, pregenual anterior cingulate cortex (pACC), and subcortical structures, including the putamen and caudate (Figures 2D–H).

### SEEG Data: Estimation of the Structures Showing High Epileptogenicity

**Figure 3:**
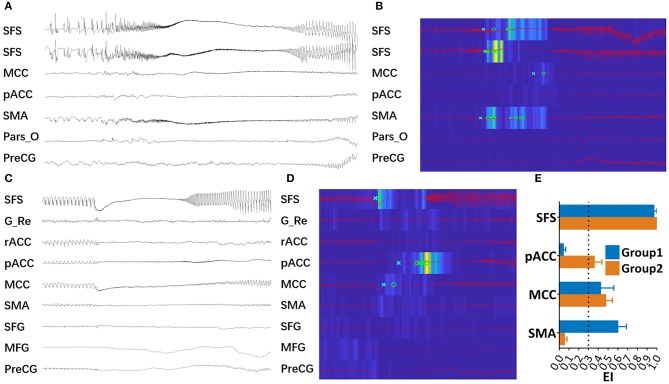
High epileptogenic structures in 2 groups. **(A)** SEEG recording of one seizure of Case 3 in group 1. **(B)** EI values of the same seizure event presents an increased energy ratio at the ictal onset (from blue to yellow scale) and the detection parameters (circle, alarm time; cross, detection time) in each channel. This map showed that SFS, SMA, and MCC were highly epileptogenic. **(C)** SEEG recording of one seizure of Case 13 in group 2. **(D)** EI values map of the same seizure attack, in which SFS, MCC, and pACC were highly epileptogenic. **(E)** Mean EI value of the SFS, pACC, MCC, and SMA in each group. The arbitrary cut-off value used to identify the extended EZ was set at 0.3, as previously described (18). (G_Re, gyrus rectus; MFG, middle frontal gyrus; Pars_O, pars opercularis; PreCG, pre-central gyrus, rACC, rostral anterior cingulate cortex, SFG, superior frontal gyrus).

The EI values are available from 9/17 patients, and all explored brain structures were computed and averaged in each case. We observed that all patients had maximal EI value within SFS sulcus, and high values of EI could also be revealed in other structures. The arbitrary cut-off value used to identify the high epileptogenicity was set at 0.3, as previously described (18). Accordingly, we found that in group 1, the SMA (0.50 ± 0.09) and MCC (0.42 ± 0.14) participated in the seizure network, and in group 2, the MCC (0.47 ± 0.07) and pACC (0.36 ± 0.08) showed a high level of epileptogenicity. Examples from the two groups are illustrated in Figure 3 (B, case 3; D, case 13). The EI values of the SFS, MCC, SMA, and pACC were obtained to demonstrate the differences between the two groups (Figure 3E).

**Figure 4:**
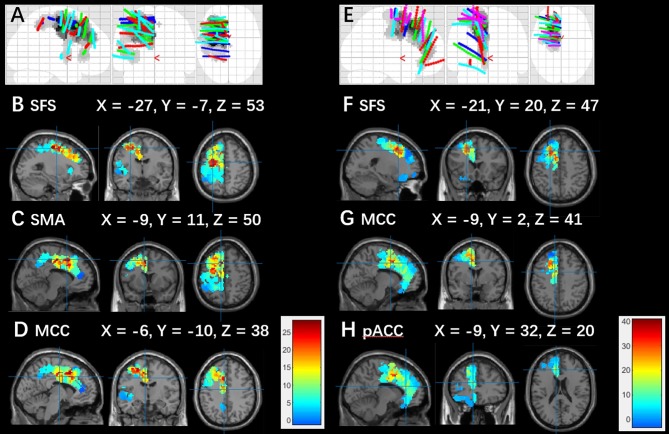
Electrodes position map and averaged epileptogenicity map. **(A)** Blue, green, red, and cyan dots show actual SEEG recording contacts from cases 1, 2, 3, and 4 in group 1. **(B–D)** EM averaged over the patients in group 1. **(E)** Blue, green, red, cyan, and pink dots indicate actual SEEG recording contacts from cases 10, 11, 12, 13, and 15 in group 2. **(F–H)** EM averaged over the patients in group 2. The significance map calculated based on the power of high frequencies at seizure onset was corrected by FWE (*p* < 0.05). The color scale indicates T scores. Electrodes and maps are drawn over the MNI152 atlas. Note that the epileptogenicity maps in patients with right-sided lesions are all horizontally flipped to the left side.

EM highlighted results similar to those from EI. For group 1, seizures originated from the SFS; subsequently, the SMA developed predominant activation, and the SMA was also involved. For group 2, in addition to the SFS, the most significant activation was found in the MCC, and a moderate activation was shown in the pACC (Figure 4).

### Location of SFS-Related Hypometabolic Regions, and High Epileptogenicity Areas

**Figure 5:**
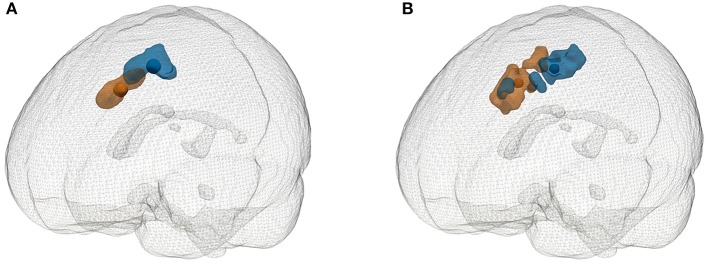
Glass brain rendering of the SFS-related hypometabolic areas (FDR-cor < 0.05) and highly epileptogenic regions (*T*-value > 25). **(A)** Significantly hypometabolic areas compared with the control and corresponding centroids. **(B)** Highly epileptogenic regions and corresponding centroids. Blue represents group 1, and orange represents group 2.

For PET analysis, we selected the SFS hypometabolism areas that survived FDR correction 0.05. Results showed that the centroid of group 2 was anterior to that of group 1, with a distance of 2.14 cm. Based on the results of EM, an SFS-related epileptic region with a T score over 25 was extracted, and a posterior-anterior positional relationship was also found. The distance between these two centroids was 2.06 cm. The results mentioned above are reported through 3D rendering in Figure 5.

## Discussion

This study demonstrated the symptomatic grouping of 17 patients with EZ confined to the SFS, which is a common site for FCD type II. Two semiology subtypes were identified. Epileptic propagation networks were delineated functionally by interictal PET in all subjects. High epileptogenic structures were analyzed by two different quantitative measures of SEEG ictal activity, EI and EM, for subjects in whom SEEG data was available.

The main findings were as follows: (I) SFS seizures can be reasonably divided into two groups of distinctive clinical features. Clear anatomical electroclinical correlations were shown, namely subjects presenting elementary motor signs showed involvement of a relatively posterior network (SFS-SMA), whereas subjects presenting complex motor behavior showed involvement of a relatively anterior network (SFS-MCC-pACC); (II) concordance was seen between the location of the SFS-related hypometabolic regions and EZs in groups 1 and 2, located along a posterior-anterior axis; and (III) according to the PET analysis, 2 different patterns of hypometabolism were seen, involving not only perilesional zones but also the primary motor cortex in group 1, and subcortical nuclei in group 2. These points are discussed in the following section. An additional observation is that good correlation was observed between the 2 different quantification methods of ictal activity on SEEG (Epileptogenicity Index and Epileptogenicity Mapping) (18, 20), to our knowledge the first study comparing these in the same patient population.

### Version and Tonic Posturing With an Underlying Network of SFS-SMA

It has long been proposed that the human frontal eye field (FEF) is located either in the vicinity of the precentral sulcus and/or in the depth of the most caudal part of the SFS (23). The FEF is activated during the initiation of eye movement, such as voluntary saccades and pursuit (24). Our patients in group 1 showed a forced and sustained head and eye version to the contralateral side, which echoed the findings of a previous study, in which versive movements were elicited by direct electrical stimulation in the posterior part of the SFS (25). The motor symptoms in this group, including the tonic of limbs and asymmetric tonic posturing, might be interpreted as the propensity for seizure propagation to the SMA (26). Our findings suggest that the posterior part of the SFS and SMA are strongly connected during seizures, which could be explained by a previous non-human primate study that showed that the frontal lobe is characterized by strong cortico-cortical connections between the lateral prefrontal cortex and SMA (27). The supplementary eye field lies in the dorsomedial premotor cortex, immediately anterior to the SMA, and involuntary version has been reported in SMA epilepsy and SMA functional mapping cases (28). The close relationship of the SFS and SMA in this cohort of patients, combined with the versive semiology, produces a strong argument for the involvement of a network controlling eye movement.

### Complex Motor Behavior With an Underlying Network of SFS-MCC-pACC

In group 2, in addition to the SFS, congruent positive results of PET/SPM and EI/EM indicating the MCC and pACC are involved in the seizure networks underlying semiological expression, which was characterized by fear, hypermotor activity and pouting. This finding is in line with a previous study from our team, which proposed that the pACC and MCC may be involved in seizure networks associated with ictal fearful expression and autonomic symptoms and that these features are common in patients with hyperkinetic motor signs (15). It has been demonstrated that both the MCC and pACC have reciprocal connections with the amygdala, which plays an essential role in the development and expression of fear (29, 30). Stimulation of the MCC has been reported to produce fear-related behavior, with moderate to excessive agitation (15). Previous studies based on fMRI have shown that the pACC is involved in the appraisal and expression of negative emotions, such as fear (31). Ictal pouting, which may be interpreted as an expression of negative emotion in some cases when associated with other behaviors (agitation, crying, or screaming for example), has been linked to dorsal anterior cingulate cortex function (32). The hyperkinetic movement observed in this group is in accordance with the previous perception that the MCC has a skeletomotor function since it projects to the striatum, pons, cerebellum, and spinal cord (29).

Epilepsy with fear and hyperkinetic behavior as the main symptoms have been previously reported in patients with EZ located in the subgenual ACC, orbitofrontal cortex, frontal pole, anterior insula, amygdala, and temporal pole (33–35). The present results reveal that fearful ictal behavior may also occur in seizures arising from the SFS, which is of relevance when planning surgical strategy. Group 1 vs. Group 2, posterior premotor vs. anterior prefrontal

Due to the expression of version, we propose that the spatial centroid of group 1 overlaps with FEF, which is mostly thought to reside at the caudal end of the superior frontal sulcus, adjacent to the premotor area (36). In group 2, we found that the anterior part of the SFS was strongly connected with the cingulate cortex. This finding is in accordance with a previous tract-tracing study, which showed a strong connection between the dorsal lateral prefrontal cortex and the cingulate cortex (37). In addition, the fear-based semiology finding could be in line with the idea that the dorsolateral prefrontal cortex participates in emotional regulation (38). Accordingly, it is reasonable to consider the anterior part of the SFS to be part of the prefrontal cortex. In our study, we found a connection between the lateral and medial regions of the frontal lobe. A previous clinical study reported a clear tendency for ictal discharge beginning in the lateral prefrontal cortex to propagate to medial structures, notably, the anterior cingulate region and the pre-SMA (5). There are also tract-tracing studies showing that multiple prefrontal and premotor regions project to the SMA and the cingulate motor areas (37, 39).

It is widely accepted that the semiologic and electrical pattern of frontal lobe seizures are difficult to characterize. The importance of our study is that we found that in the frontal lobe, even within a single sulcus, the EZ differs within a centimeter range, with different epilepsy network organization and distinct clinical patterns. These patterns could probably be explained by the developmental origin of FCD within caudal premotor and rostral dorsolateral prefrontal areas, respectively.

### Interictal Hypometabolic Involvement of Primary Motor Areas and Subcortical Structures in the 2 Groups

All of our cases were FCD type II, and according to previous reports, it might have been expected that the maximally hypometabolic region would be limited to the peri-lesional area (40). However, there was a wide range of hypometabolic areas around the SFS in group 1, which affected the primary motor cortex. This finding may be explained by the fact that epileptic discharge propagation reached this area. Previous study in non-human primates has shown that the dorsal part of the premotor area and the primary motor area share a common network and are activated in a parallel manner (41).

It is of interest that in group 2, the caudate and putamen showed significant hypometabolism. This finding may be interpreted as the epileptic network affecting the cortical-subcortical circuitry. The rostral striatum, including the caudate and putamen, receives projections from the prefrontal cortex and the ACC; together, these structures drive actions, including emotions, motivation, and cognition (42). Our findings echo a recent report of cortical-striatal circuitry in a patient with frontal epilepsy, in which the caudate was capable of generating spontaneous and stimulation-induced seizures (43).

### Limitation

Our results were presented in EI, EI-map, and SPM-PET. Although the consistency between the results is acceptable, however, the EI and EM are based on the contacts of the SEEG; therefore, the spatial resolution is limited, and sampling bias may exist. According to the results from the EI and EI-map, the MCC might be involved in Group 1. However, the SMA and MCC are closely adjacent, so we cannot exclude that there may be a sampling bias here. SPM-PET is a whole-brain analysis method, by which we found that the involvement of SMA is more significant compared to MCC. When we were using a more rigorous significance level of SPM-PET (FDR = 0.01), SMA had a significant 499 voxels, and the MCC did not exceed 50 voxels. More importantly, the prominent semiology, version, and tonic posturing support the SMA as mentioned above. When the MCC was regarded as the semiology responsible area, as previous studies described, these patients supposed to reveal predominantly complex motor behavior or hypermotor (9, 44, 45). Interestingly, we observed these symptoms pattern in another group. In addition to the sampling bias, the sample size of SEEG patients in the study was small, and one of the patients did not achieve seizure-free. We think this was related to incomplete resection. To further solve the above problems, future studies with a larger sample size and more sophisticated design (such as mapping) are needed.

## Conclusion

We have identified two distinct semiology subtypes in patients with seizures originating from the SFS with multimodal evidence. For patients with elementary motor signs (version and tonic posturing), the posterior SFS-SMA network plays a crucial role. On the other hand, for patients with complex motor behavior (fear, hypermotor activity, and pouting), the anterior SFS-MCC-pACC and the subcortical nuclei appears to be the underlying network. Such results advance knowledge on frontal lobe epilepsy networks, with implications for optimal presurgical localization of frontal lobe epilepsy.

## Data Availability Statement

The datasets generated for this study are available on request to the corresponding author.

## Ethics Statement

The studies involving human participants were reviewed and approved by The Ethics Board of Beijing Tiantan Hospital. Written informed consent to participate in this study was provided by the participants' legal guardian/next of kin. Written informed consent was obtained from the individual(s), and minor(s)' legal guardian/next of kin, for the publication of any potentially identifiable images or data included in this article.

## Author Contributions

KZ guided the design of the study, performed surgical resection, reviewed image data. CZ and BZ designed the study, collected and analyzed imaging/functional data, and drafted manuscript. AM provided conceptual discussion and revised the manuscript. WH wrote analyzing code and reviewed image data and SEEG data. XW and YM reviewed clinical data. XS reviewed clinical and EEG/SEEG data. JZ reviewed image data and revised the manuscript.

### Conflict of Interest

The authors declare that the research was conducted in the absence of any commercial or financial relationships that could be construed as a potential conflict of interest.

## Supplementary Material

Figure S1 Surgical plans for 17 patients.
