# Supplementary figures and images for: Superior Frontal Sulcus Focal Cortical Dysplasia Type II: An MRI, PET, and Quantified SEEG Study
Source: Front Neurol. 2019 Dec 3;10:1253. doi: 10.3389/fneur.2019.01253 (PMC6915108; doi:10.3389/fneur.2019.01253)

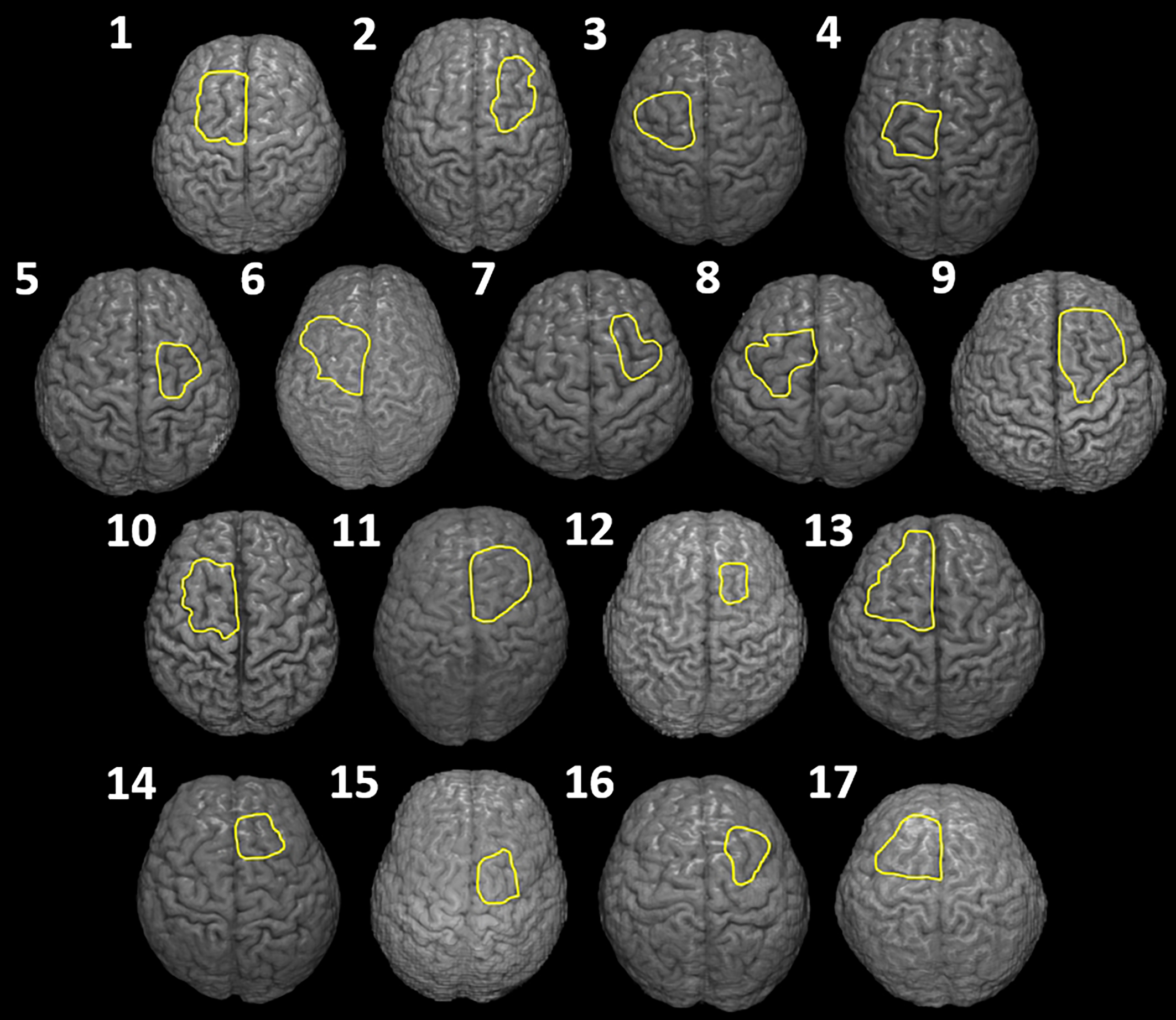

Supplement: Figure S1 — Surgical plans for 17 patients. [file Image_1.TIF]
